# Can Diagnostic Delay Amplify Drug Resistance in Tuberculosis? A Critical Integrative Reflection on Bacterial Adaptation, Persistence, Within-Host Evolution, and Heteroresistance

**DOI:** 10.3390/diseases14090344

**Published:** 2026-09-17

**Authors:** Ariel Torres, Paloma González, Gisselle Trujillo, Martha Fors

**Affiliations:** 1 Facultad de Ciencias de la Salud y Bienestar Humano, Universidad Tecnológica Indoamérica, Quito 170301, Ecuador; 2 Biblioteca, Universidad de Las Américas, Quito 170124, Ecuador; 3 One Health Research Group, Facultad de Ciencias de la Salud, Universidad de Las Américas, Quito 170124, Ecuador

**Keywords:** tuberculosis, drug resistance, diagnostic delay, within-host evolution, heteroresistance

## Abstract

Background/Objectives: Drug resistance in tuberculosis remains one of the major challenges to global disease control. Traditionally, its emergence has been attributed to factors such as treatment interruption, poor adherence, and the selective pressure exerted by antimicrobial agents. However, recent advances in bacterial genetics, within-host evolution, cellular persistence, and heteroresistance suggest that relevant biological processes may develop before treatment initiation. The aim of this critical integrative reflection was to explore the biological plausibility that diagnostic delay may act as an amplifying factor for adaptive and evolutionary mechanisms potentially associated with drug resistance in Mycobacterium tuberculosis. Methods: A structured documentary search was conducted in PubMed/MEDLINE, Scopus, Web of Science, and Google Scholar. Of the 86 documents initially identified, 29 studies were selected using predefined inclusion and exclusion criteria. The evidence was analysed through a conceptual convergence matrix, which enabled the organisation of findings into five analytical categories: diagnostic delay and programmatic determinants; mechanisms of resistance, bacterial adaptation, and clinical implications; persistence and drug tolerance; within-host evolution and genetic diversity; and heteroresistance and resistant subpopulations. In addition, four institutional reports were incorporated to contextualise the issue from a global perspective. Results: The reviewed evidence suggests that extended persistence of infection before diagnosis may favour biological opportunities for bacterial adaptation, the persistence of tolerant subpopulations, the accumulation of genetic diversity, and the emergence of variants with distinct drug susceptibility profiles. Although the available studies do not demonstrate a direct causal relationship, they support the plausibility of an evolutionary trajectory capable of influencing the dynamics of drug resistance. Conclusions: An expanded model of drug resistance in tuberculosis is proposed, in which diagnostic delay does not constitute a direct cause of resistance but rather a potential amplifying factor for biological adaptation and evolutionary change occurring before treatment initiation. This hypothesis generates new avenues for research on the interaction between timely diagnosis, bacterial evolution, and drug resistance.

## 1. Introduction

Tuberculosis remains one of the most important infectious diseases worldwide and continues to represent a persistent challenge for health systems, particularly in low- and middle-income countries [1]. According to the World Health Organization [2], an estimated 10.8 million new cases of tuberculosis and 1.25 million tuberculosis-related deaths occurred in 2023, maintaining the disease as the leading cause of death from a single infectious agent globally. Despite the diagnostic and therapeutic advances achieved over recent decades, the burden of disease remains substantial and is disproportionately concentrated among socially vulnerable populations with limited access to timely healthcare services.

One of the major obstacles to effective tuberculosis control is the persistence of diagnostic gaps. The WHO [2,3] has repeatedly reported that millions of individuals with tuberculosis remain undiagnosed or experience significant delays between symptom onset and treatment initiation. These delays not only facilitate community transmission and clinical deterioration but also reflect structural limitations related to access to healthcare services, diagnostic capacity, early case detection, and the programmatic response of health systems. In the region of the Americas, the Pan American Health Organization [4] has warned that delayed case identification continues to constitute a major barrier to achieving the targets established under the End TB Strategy.

At the same time, drug-resistant tuberculosis has emerged as one of the greatest threats to global tuberculosis control programmes. According to the WHO [2], hundreds of thousands of individuals develop rifampicin-resistant tuberculosis or multidrug-resistant tuberculosis each year, conditions associated with higher healthcare costs, longer treatment regimens, and lower probabilities of treatment success. International reports have consistently emphasised that drug resistance is a complex phenomenon influenced by microbiological, clinical, social, and programmatic factors [2,3,4]. Traditionally, explanations have focused on the selective pressure generated by inappropriate antimicrobial use (particularly fluoroquinolones and aminoglycosides), inadequate treatment, treatment interruption, or poor adherence; however, important questions remain regarding other mechanisms that may contribute to its development.

Within this context, it is pertinent to explore whether diagnostic delay may play a broader role than has traditionally been recognised. Beyond its epidemiological and clinical consequences, the prolonged period between the onset of active disease and treatment initiation may represent a biological window during which adaptive and evolutionary processes could potentially occur. Although such processes have been documented in *Mycobacterium tuberculosis* infection, available evidence does not establish that their occurrence or magnitude increases as a direct consequence of diagnostic delay.

## 2. Methodology

A critical integrative reflection with an analytical and hypothesis-generating approach was conducted to examine the biological plausibility of an expanded relationship between diagnostic delay and the evolution of drug resistance in tuberculosis. The analysis was based on the conceptual integration of evidence from different fields of research, including epidemiology, microbiology, bacterial genetics, and within-host evolution.

The aim was not to quantify associations through statistical synthesis or to establish definitive causal relationships, but rather to critically integrate evidence from diverse research domains in order to develop a conceptual interpretation capable of generating new scientific hypotheses.

The literature search was conducted between March and May 2026 using the PubMed/MEDLINE, Scopus, Web of Science, and Google Scholar databases. Combinations of terms related to tuberculosis, diagnostic delay, and biological mechanisms associated with drug resistance were employed, including tuberculosis, *Mycobacterium tuberculosis*, diagnostic delay, delayed diagnosis, drug resistance, drug-resistant tuberculosis, drug tolerance, persistence, persister cells, within-host evolution, genetic diversity, heteroresistance, adaptive evolution, host–pathogen interaction, and bacterial adaptation. The search strategy prioritised publications from the period 2020–2026; however, earlier studies considered fundamental for understanding processes related to within-host evolution, genetic diversity, and bacterial adaptation were also included when their conceptual relevance justified inclusion.

The initial search identified 86 potentially relevant documents. Subsequently, a sequential review of titles, abstracts, and full-text articles was conducted to determine their relevance to the study objective. Eligible publications included original observational and experimental studies related to tuberculosis and drug resistance, microbiological, genomic, molecular, and evolutionary investigations of *Mycobacterium tuberculosis*, studies addressing diagnostic delay and programmatic determinants, as well as research focused on bacterial adaptation, persistence, drug tolerance, within-host evolution, genetic diversity, and heteroresistance.

Reviews providing biological or conceptual foundations directly related to the proposed hypothesis were also considered. Duplicate records, case reports, editorials, commentaries, letters to the editor, and publications lacking sufficient methodological development were excluded. Studies focused exclusively on pharmacological efficacy without addressing biological mechanisms of resistance, as well as publications that did not provide evidence relevant to the predefined analytical domains, were also excluded. Following this selection process, 29 studies were retained because of their contribution to understanding the mechanisms potentially involved in the evolution of drug resistance.

Studies were retained on the basis of their relevance to the research question and the predefined analytical domains of diagnostic delay, bacterial adaptation, persistence, within-host evolution, heteroresistance, and drug resistance. Evidence was considered according to its contribution to examining these domains, irrespective of whether the findings supported the proposed conceptual hypothesis.

The selected studies were incorporated into a documentary analysis matrix specifically designed for this investigation. For each publication, information was extracted regarding the study objective, methodological design, biological mechanisms described, principal findings, implications for drug resistance, and potential links to the proposed conceptual hypothesis. The evidence was critically appraised in relation to study design, methodological development, relevance to the analytical domains, and consistency between the reported findings and the interpretations incorporated into the conceptual synthesis.

Subsequently, a comparative and integrative analysis was undertaken to identify recurring patterns, conceptual relationships, and convergent biological mechanisms. Unlike qualitative approaches based on theoretical saturation, the present study employed a conceptual convergence matrix, the purpose of which was to identify complementary mechanisms described across different lines of research and to explore their potential integration within a common evolutionary pathway.

**Figure 1:**
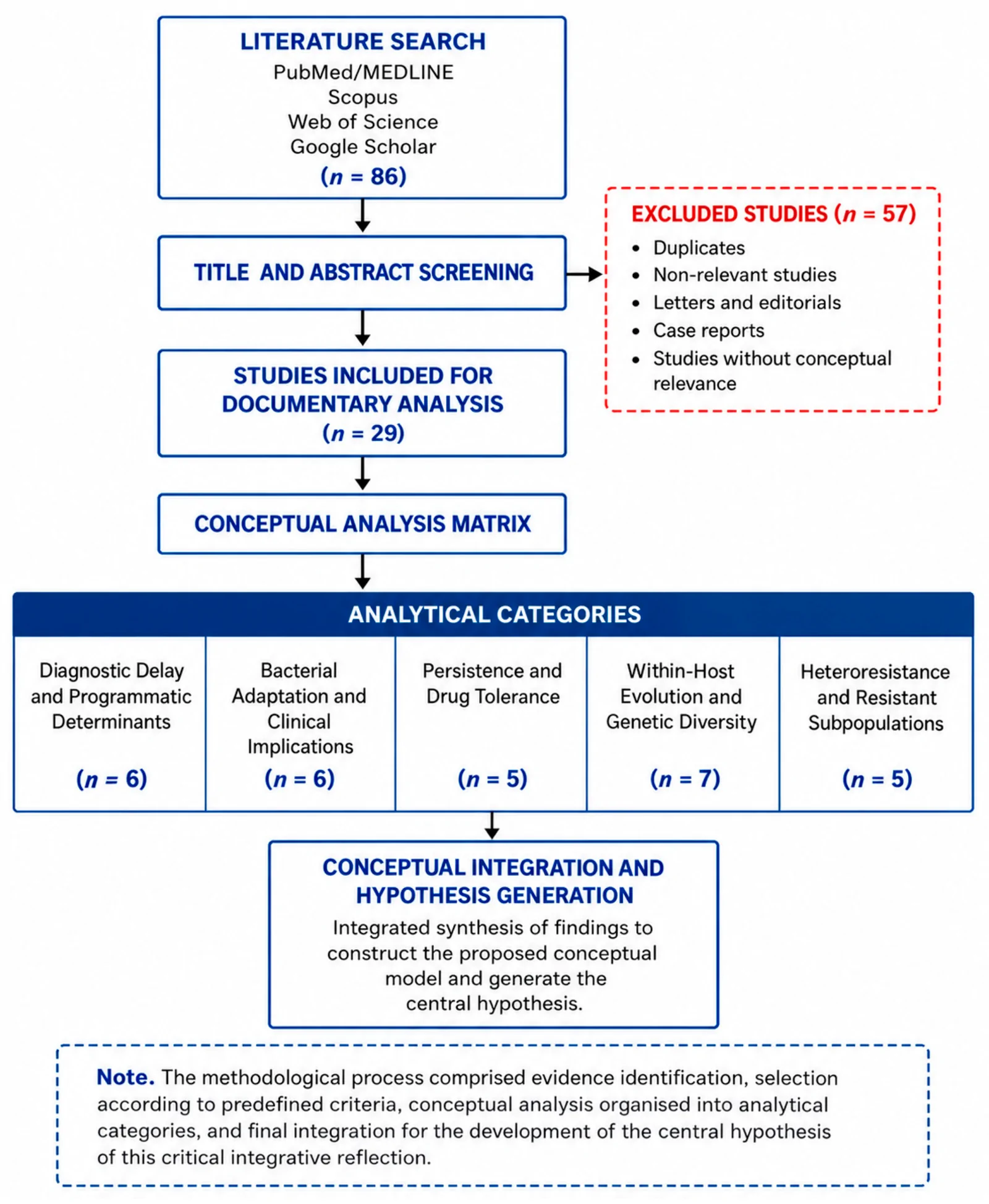
Methodological Flowchart of the Critical Integrative Reflection and Conceptual Analysis Process.

This process enabled the organisation of the evidence into five interrelated analytical categories: diagnostic delay and programmatic determinants (6 studies); mechanisms of resistance, bacterial adaptation, and clinical implications (6 studies); persistence and drug tolerance (5 studies); within-host evolution and genetic diversity (7 studies); and heteroresistance and resistant subpopulations (5 studies). These categories constituted the foundation for the synthesis of findings and for the development of the conceptual framework presented in this critical integrative reflection (Figure 1).

The emerging conceptual hypothesis was developed through the convergence of evidence from multiple disciplines, including epidemiology, microbiology, bacterial genetics, within-host evolution, and antimicrobial pharmacology. The objective was not to establish causality but rather to explore the biological plausibility of an expanded interpretation of drug resistance in tuberculosis, in which diagnostic delay may act as an amplifying factor for adaptive and evolutionary processes occurring before treatment initiation.

This study was based exclusively on published scientific literature and publicly accessible documents. It did not involve human participants, experimental animals, or identifiable personal data and therefore did not require approval from a research ethics committee or informed consent.

Generative artificial intelligence tools were used exclusively to support language editing, grammatical revision, preliminary content organisation, and the development of the conceptual figures included in the manuscript. Literature selection, critical appraisal of the evidence, development of the analytical categories, formulation of the conceptual hypothesis, and final manuscript preparation were performed and verified entirely by the authors. Artificial intelligence was not involved in the scientific interpretation of the findings or in the autonomous generation of conclusions.

## 3. Results

The analysis of the selected studies enabled the identification of biological, clinical, and programmatic processes potentially relevant to the proposed hypothesis on drug resistance in tuberculosis. The findings were organised into five interrelated analytical categories representing distinct but potentially interconnected domains of evidence, rather than an empirically demonstrated sequential pathway.

The first category addressed diagnostic delay and the programmatic determinants associated with delayed case detection and treatment initiation. The second examined mechanisms of bacterial adaptation and host–pathogen interactions that promote the survival of *Mycobacterium tuberculosis*. The third explored the phenomena of persistence and drug tolerance, whereas the fourth analysed within-host evolution and the generation of genetic diversity. Finally, the fifth category synthesised evidence on heteroresistance and resistant subpopulations as potential intermediate stages in the consolidation of drug resistance.

Taken together, these categories suggest that drug resistance may be interpreted as the outcome of multiple dynamic processes that interact throughout the natural history of tuberculosis infection.

### 3.1. Diagnostic Delay and Programmatic Determinants

The studies analysed consistently identified diagnostic delay as a relevant determinant of clinical and programmatic tuberculosis outcomes. Several investigations demonstrated that delays in seeking medical care, diagnostic confirmation, or treatment initiation are associated with a higher likelihood of treatment failure, mortality, loss to follow-up, and reduced treatment success [5,6,7,8].

Multiple factors contributing to these delays were also identified, including barriers to healthcare access, a low level of clinical suspicion, diagnostic limitations, adverse socioeconomic conditions, population mobility, and structural weaknesses within health systems [5,9,10]. In particularly vulnerable settings, such as prison systems, these limitations are further exacerbated by overcrowding, insufficient epidemiological surveillance, and difficulties in ensuring timely access to diagnosis and treatment [10].

Taken together, the available evidence suggests that diagnostic delay not only facilitates disease transmission but also prolongs the duration of active infection before treatment initiation [5,6,7,8,9,10].

### 3.2. Mechanisms of Resistance, Bacterial Adaptation, and Clinical Implications

The prolonged duration of active infection before treatment initiation provides a biological framework for exploring the mechanisms that promote the survival and adaptation of *Mycobacterium tuberculosis* within the host. The studies included in this category indicate that *Mycobacterium tuberculosis* possesses multiple strategies that facilitate persistence within the host, including metabolic adaptation, intracellular survival, modulation of the immune response, and the establishment of biological niches that favour extended persistence of infection [11,12,13].

Furthermore, specific bacteriological characteristics and genomic profiles have been associated with more prolonged bacillary persistence and less favourable treatment responses, suggesting the existence of adaptive mechanisms that directly influence the clinical evolution of the disease [14]. From a population perspective, the expansion of drug-resistant tuberculosis also depends on the interaction between bacterial adaptation, community transmission, and epidemiological factors that facilitate the circulation of resistant strains [15].

Although therapeutic advances have improved the management of drug-resistant tuberculosis, important differences in the effectiveness and safety of available treatment regimens persist, reflecting the biological and clinical complexity of this phenomenon [16]. Taken together, these findings suggest that bacterial adaptation is a dynamic process that facilitates the persistence of infection within the within-host microenvironment [11,12,13,14,15,16].

### 3.3. Persistence and Drug Tolerance

The studies analysed consistently indicate that certain bacterial subpopulations are capable of surviving prolonged exposure to antimicrobial agents without necessarily developing classical resistance-conferring mutations. Because much of this evidence derives from antimicrobial exposure, it supports the biological basis of persistence and drug tolerance but cannot be directly extrapolated to the pre-treatment period considered in the present hypothesis. This survival is mediated through adaptive physiological states characterised by metabolic downregulation, stress responses, and the formation of persister cells [17,18].

Furthermore, drug tolerance has been shown to evolve dynamically during infection and treatment, although evidence involving adequate drug exposure specifically reflects treatment-associated processes [19]. Several studies suggest that these persistent populations play an important role in prolonging infection, contributing to relapse and unfavourable treatment outcomes, and potentially serving as reservoirs from which resistant variants may subsequently be selected under therapeutic pressure [20].

In addition, the capacity for drug tolerance does not appear to be uniformly distributed across *Mycobacterium tuberculosis* strains but may vary according to specific phylogenetic characteristics, suggesting intrinsic differences in the ability to survive pharmacological pressure during treatment [21]. Taken together, these findings indicate that persistence and drug tolerance represent biological mechanisms that prolong the within-host survival of the microorganism, creating favourable conditions for the progressive accumulation of evolutionary changes that are explored in the subsequent category addressing genetic diversity and within-host evolution [17,18,19,20,21].

### 3.4. Within-Host Evolution and Genetic Diversity

Once the capacity of *Mycobacterium tuberculosis* to persist for prolonged periods within the host has been established, the available evidence suggests that this prolonged persistence of infection favours ongoing processes of genetic evolution and population diversification. The studies analysed consistently demonstrate that bacillary populations do not remain static during infection but instead undergo dynamic genomic changes associated with biological adaptation, alterations in the within-host microenvironment, and, in treatment-related observations, therapeutic pressure [22,23,24].

Several investigations have shown that the progressive accumulation of mutations, the emergence of minor variants, and the coexistence of multiple bacterial subpopulations may occur even in initially drug-susceptible strains, generating heterogeneous treatment responses and promoting complex evolutionary trajectories within the host [25,26]. Longitudinal studies have further demonstrated that *Mycobacterium tuberculosis* populations undergo continuous changes over time, including the expansion, replacement, or disappearance of genetic variants, reflecting dynamic within-host evolutionary processes over the periods observed [27].

Moreover, the available evidence indicates that specific phylogenetic characteristics may influence the adaptive capacity of the bacillus and its propensity to develop drug resistance, suggesting that genetic diversity constitutes a central component in the evolution of resistant strains [28]. Taken together, these findings indicate that within-host evolution represents a dynamic and continuous process that progressively increases the genetic diversity of *Mycobacterium tuberculosis* [22,23,24,25,26,27,28].

### 3.5. Heteroresistance and Resistant Subpopulations

Given the existence of dynamic genetic diversity within the host, the available evidence indicates that one of its most relevant manifestations is the emergence of heteroresistance and the formation of bacterial subpopulations with distinct drug susceptibility profiles. The studies analysed consistently indicate that heteroresistance represents the simultaneous coexistence of susceptible and resistant variants within a single *Mycobacterium tuberculosis* population, constituting an intermediate stage between complete drug susceptibility and fully established drug resistance [29,30].

Furthermore, several investigations have demonstrated that these subpopulations may arise as a consequence of within-host evolution, the progressive accumulation of mutations, and, in some cases, the simultaneous coexistence of different infecting strains within the same host [31]. The available evidence also suggests that many of these resistant variants initially remain at low frequencies and may escape detection by conventional diagnostic methods, subsequently expanding under pharmacological pressure and promoting progression towards more stable forms of drug resistance [29,32].

From a clinical perspective, heteroresistance has been associated with a higher likelihood of treatment failure, persistence of infection, and reduced treatment success, highlighting its prognostic and therapeutic significance [33]. Taken together, these findings suggest that heteroresistance represents a dynamic evolutionary phenomenon linking within-host genetic diversity to the emergence of clinically detectable drug resistance, through which prolonged processes of adaptation and selection may ultimately culminate in established forms of drug-resistant tuberculosis [29,30,31,32,33].

### 3.6. Synthesis of Findings

Taken together, the analysed evidence identifies two complementary but distinct domains: evidence that diagnostic delay and programmatic limitations prolong the duration of active infection before treatment initiation, and evidence that *Mycobacterium tuberculosis* can undergo bacterial adaptation, persistence, within-host evolution, genetic diversification, and heteroresistance. The potential connection between these domains constitutes the central hypothesis of the present conceptual model; however, the reviewed studies do not directly demonstrate that a longer diagnostic delay increases the occurrence or magnitude of these evolutionary processes.

Although the studies reviewed do not demonstrate a direct causal relationship between diagnostic delay and the development of drug resistance, the findings highlight multiple biologically plausible mechanisms that may act as amplifying factors within the proposed conceptual framework (Table 1).

## 4. Critical Reflection

Traditionally, diagnostic delay in tuberculosis has been interpreted primarily as an indicator of health system performance and as a determinant of community transmission, morbidity, mortality, and unfavourable treatment outcomes [9,10]. However, the findings identified in this review suggest an additional dimension that has received comparatively limited attention. Beyond its epidemiological and clinical implications, each additional week or month during which *Mycobacterium tuberculosis* remains within the host represents a biological window in which the bacillus continues to interact with the immune system, adapt to changing environmental conditions, and maintain active processes of survival and replication.

Although this interpretation does not imply a direct causal relationship between diagnostic delay and drug resistance, the available evidence supports exploring the possibility that prolonged pre-treatment infection may provide additional time during which bacterial adaptation, persistence, and genetic diversification can occur. Whether diagnostic delay increases the frequency or extent of these processes, or contributes to the subsequent emergence of resistant subpopulations, remains to be directly demonstrated.

Mechanisms of bacterial adaptation help explain why diagnostic delay should not be viewed solely as a healthcare delay but also as an extension of the biological period during which *Mycobacterium tuberculosis* actively interacts with the host. During this interval, the bacillus may modulate immune responses, adapt to adverse metabolic conditions, survive within intracellular niches, and maintain persistent bacillary populations [11,12,13]. This adaptive capacity suggests that the natural history of tuberculosis should not be interpreted as a passive pre-treatment phase but rather as a dynamic process in which the microorganism accumulates survival advantages over time.

**Figure 2:**
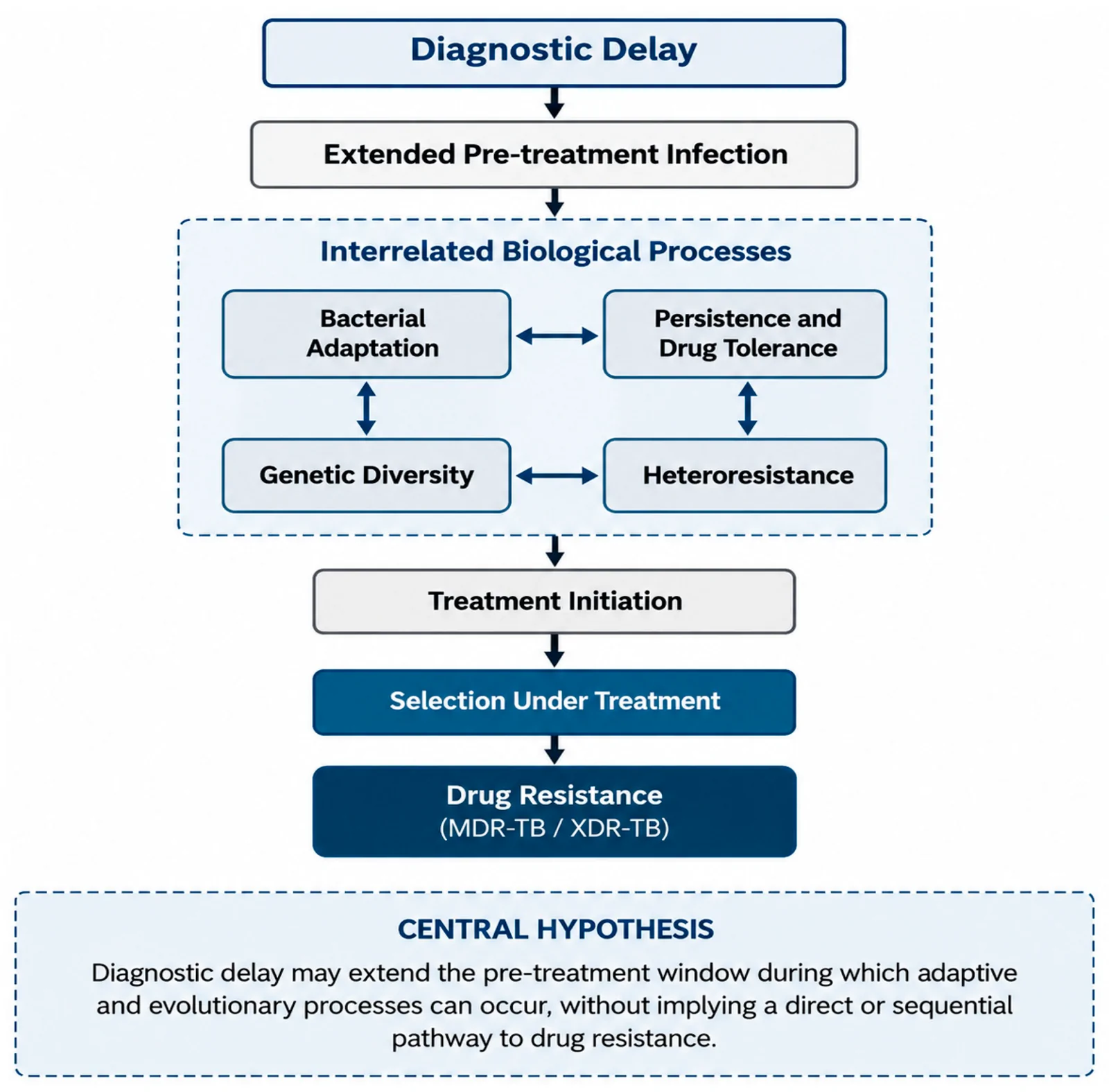
Expanded conceptual model of drug resistance in tuberculosis. The biological processes represented are potentially interrelated and overlapping and should not be interpreted as a fixed sequential or causal pathway.

In this regard, the findings reported by Pang et al. [14] and Che et al. [15] suggest that the bacteriological, genomic, and epidemiological characteristics of the bacillus may influence both clinical persistence and the transmission of resistant strains. Consequently, the expanded model incorporates host–pathogen adaptation as a potentially relevant biological process during prolonged infection, while recognising that its proposed relationship with diagnostic delay, persistence, drug tolerance, and genetic diversification remains conceptual rather than empirically established (Figure 2).

Importantly, the stages incorporated into the proposed model should not be interpreted as strictly sequential biological events. Rather, they represent interconnected and potentially overlapping processes that may interact dynamically throughout the course of infection. Adaptation, persistence, genetic diversification, and heteroresistance are likely to influence one another through complex feedback mechanisms, contributing collectively to the evolutionary trajectory of *Mycobacterium tuberculosis* within the host.

If host–pathogen adaptation provides the conditions necessary for the prolonged survival of *Mycobacterium tuberculosis*, the phenomena of persistence and drug tolerance represent the functional expression of this adaptation. The studies analysed suggest that a proportion of the bacillary population may remain viable for extended periods through physiological states characterised by low metabolic activity, adaptive stress responses, and the formation of persister cells [17,18]. Although these mechanisms do not constitute genetic resistance in the strict sense, they enable the survival of bacterial subpopulations under adverse conditions, including exposure to antimicrobial agents [19].

These findings raise an important question: the longer the bacillus remains within the host, the greater the opportunities for these persistent populations to survive, adapt, and maintain active biological reservoirs. Consequently, persistence may represent one of the biological processes potentially interacting with bacterial adaptation and subsequent evolutionary dynamics. This interpretation is consistent with the proposal advanced by Negi and Sharma [20], who suggest that persister cells may constitute niches from which better-adapted variants subsequently emerge. Therefore, rather than representing an isolated phenomenon, drug tolerance may constitute one of several interrelated biological processes potentially relevant to the evolutionary dynamics associated with the emergence of drug resistance.

Once prolonged survival of *Mycobacterium tuberculosis* within the host is considered, time may represent a biologically relevant context for evolutionary processes. The studies included in this category demonstrate that bacillary populations can undergo genetic change during infection, including the accumulation of mutations, emergence of minor variants, and development of within-host genetic diversity [22,23,24]. These findings establish the occurrence of within-host evolutionary processes but do not determine whether their extent increases with the duration of untreated infection. The findings reported by Wang et al. [26], Xu et al. [27], and Chen et al. [25] suggest that even initially drug-susceptible strains may follow complex evolutionary trajectories characterised by the emergence, expansion, or disappearance of genetic variants over time.

This observation is particularly relevant to the proposed hypothesis, although evidence of within-host genetic diversification, particularly when derived from treatment-associated observations, cannot by itself establish the extent of this process before treatment initiation. Although the available evidence does not demonstrate a direct causal relationship between diagnostic delay and drug resistance, it supports the biological plausibility that prolonged infection may favour a more complex evolutionary environment, capable of generating progressively more heterogeneous bacterial populations that are potentially better adapted to survive within the host.

Heteroresistance may be interpreted as one of the most visible manifestations of the evolutionary processes occurring within the host during tuberculosis infection. The studies analysed demonstrate that *Mycobacterium tuberculosis* populations are not necessarily homogeneous but may simultaneously contain both drug-susceptible and drug-resistant variants, even before resistance is detected using conventional diagnostic methods [29,30]. Conceptually, this phenomenon represents a point of convergence between the genetic diversity accumulated during infection and the selection processes that subsequently occur under therapeutic pressure. The findings reported by Vallejos-Sanchez et al. [32] and Yang et al. [31] suggest that resistant variants may initially emerge as minor subpopulations and remain concealed within a predominantly susceptible population, with their subsequent expansion potentially occurring under therapeutic selective pressure.

The association between heteroresistance and poorer clinical outcomes [33] further reinforces the clinical significance of this phenomenon. The reviewed evidence establishes that heteroresistance and resistant minor variants can occur within heterogeneous *Mycobacterium tuberculosis* populations; however, it does not establish that their emergence is driven by diagnostic delay. Within the proposed framework, their potential presence before treatment initiation provides a biologically plausible basis for investigating whether the duration of untreated infection influences the subsequent evolutionary dynamics of drug resistance.

Within this context, the duration of undiagnosed infection may be considered a temporal factor of potential evolutionary relevance. A longer pre-treatment period could theoretically provide additional opportunities for the survival of persistent subpopulations, genetic diversification, and the emergence of heteroresistance; however, whether these processes increase as a function of diagnostic delay has not been directly established.

Subsequently, once treatment has been initiated, these populations may be better positioned to respond to pharmacological pressure, facilitating the expansion of pre-existing resistant variants. Accordingly, an expanded model is proposed in which diagnostic delay does not act as a direct cause of drug resistance but rather as a potential amplifying factor that prolongs the temporal window during which biological and evolutionary processes associated with its emergence may occur.

If the proposed hypothesis is correct, its implications may extend beyond microbiology and directly influence the field of public health. Traditionally, strategies aimed at reducing diagnostic delay have been justified by their capacity to decrease transmission, reduce morbidity and mortality, and improve treatment outcomes. However, the evidence integrated in this reflection suggests a potential additional dimension: reducing the time elapsed between symptom onset and diagnosis may limit the biological window during which processes of bacterial adaptation, persistence, genetic diversification, and the emergence of resistant subpopulations could potentially occur.

From this perspective, early detection may represent not only an epidemiological strategy but also a potentially relevant intervention for limiting the temporal window available for bacterial evolutionary processes. Furthermore, if future investigations confirm this hypothesis, symptom duration may acquire value as a biological risk marker, enabling the identification of patients with prolonged diagnostic delays who could benefit from early drug susceptibility testing and expanded molecular investigation at the time of diagnosis.

Future research should evaluate this hypothesis through longitudinal cohort studies integrating precise measurements of diagnostic delay, serial genomic sequencing, assessment of baseline heteroresistance, and treatment outcomes. Such approaches may help clarify whether prolonged pre-treatment infection is associated with increased genetic diversification, the emergence of resistant subpopulations, or a greater probability of subsequent drug resistance development.

The implications of this hypothesis are not restricted to the microbiological or clinical domains but also extend to the programmatic and community dimensions of tuberculosis control. Insufficient knowledge of tuberculosis symptoms, the normalisation of prolonged cough, disease-related stigma, and inaccurate perceptions of risk may all contribute substantially to diagnostic delay. Consequently, strategies addressing these determinants, including targeted health education, systematic screening of populations at increased risk, strengthened clinical suspicion at the primary-care level, and improved access to rapid molecular diagnosis, could promote more timely diagnosis and thereby shorten the pre-treatment period available for bacterial adaptation and evolutionary change.

Importantly, the proposed framework should be interpreted as a hypothesis-generating conceptual model rather than as evidence of a causal pathway. Its primary purpose is to integrate currently available evidence into a coherent biological perspective capable of informing future experimental, genomic, and longitudinal investigations.

Although this hypothesis requires further validation, it offers an innovative perspective through which health education, health literacy, and early detection may be incorporated as indirect components of strategies aimed at preventing the emergence of drug resistance in tuberculosis.

## 5. Limitations

This study has limitations inherent to its nature as a critical integrative reflection. First, it is based exclusively on previously published evidence and does not incorporate primary data; therefore, it does not permit the establishment of causal relationships between diagnostic delay and the development of drug resistance in tuberculosis. Second, the included studies were conducted across different epidemiological settings, populations, and methodological designs, limiting the direct comparability of their findings. In addition, the hypothesis-driven selection and integration of evidence may introduce confirmation bias, despite consideration of findings irrespective of whether they supported the proposed conceptual hypothesis.

Furthermore, the analytical categories identified represent a conceptual integration of mechanisms described in the literature rather than an experimentally demonstrated biological sequence. Finally, although the proposed hypothesis integrates evidence derived from microbiology, bacterial evolution, genetics, and epidemiology, its validation requires longitudinal, genomic, and clinical studies specifically designed to evaluate the potential contribution of diagnostic delay to the adaptive and evolutionary processes of *Mycobacterium tuberculosis*.

## 6. Conclusions

The evidence analysed suggests that diagnostic delay may play a more complex role in the natural history of tuberculosis than has traditionally been recognised. Beyond its established epidemiological and clinical consequences, prolonged infection before treatment initiation provides an extended biological window in which bacterial adaptation, persistence, genetic diversification, and heteroresistance warrant further investigation.

Based on the integration of five interrelated analytical categories, an expanded model of drug resistance in tuberculosis is proposed, in which diagnostic delay does not constitute a direct cause of drug resistance but rather a potential amplifying factor for biological mechanisms that may subsequently facilitate the selection and expansion of resistant variants under therapeutic pressure.

If this hypothesis is confirmed by future research, its implications may extend beyond the biological understanding of drug resistance. The interval between symptom onset and diagnosis may acquire value as an indirect marker of the evolutionary risk of the bacillus and contribute to the early identification of patients with a higher likelihood of harbouring heteroresistance or emerging drug resistance. From this perspective, early detection may represent not only a strategy for reducing transmission and improving clinical outcomes but also a potentially relevant intervention for shortening the pre-treatment window considered in the proposed evolutionary framework.

## Figures and Tables

**Table 1:** Synthesis of the analytical categories and biological mechanisms incorporated into the expanded conceptual model of drug resistance in tuberculosis.

Analytical Category	Main Findings	Contribution to the Conceptual Model
Diagnostic Delay and Programmatic Determinants (*n* = 6)	Diagnostic delays are associated with poorer clinical outcomes, ongoing transmission, and prolonged infection.	Prolongs the duration of active infection before diagnosis and treatment initiation, thereby extending the pre-treatment biological window.
Bacterial Adaptation and Clinical Implications (*n* = 6)	*Mycobacterium tuberculosis* develops mechanisms of intracellular survival, metabolic adaptation, and immune evasion.	Promotes infection persistence and prolonged host–pathogen interactions.
Persistence and Drug Tolerance (*n* = 5)	Persistent bacterial subpopulations can survive adverse conditions and, under treatment, antimicrobial exposure.	Maintains viable bacterial reservoirs with adaptive potential.
Within-Host Evolution and Genetic Diversity (*n* = 7)	Mutations, minor variants, and genetic diversity have been documented during within-host infection, including treatment-associated observations.	Generates the evolutionary substrate for the emergence of variants with differing adaptive capacities.
Heteroresistance and Resistant Subpopulations *(n* = 5)	Susceptible and resistant variants can coexist within heterogeneous bacterial populations.	Represents a state of bacterial population heterogeneity potentially relevant to the emergence of drug resistance.

Note. Developed by the authors based on the analysed evidence (*n* = 29 studies).

## Data Availability

No new data were created or analysed in this study. Data sharing is not applicable to this article.
